# Development and evaluation of the Ingwavuma receptive vocabulary test: A tool for assessing receptive vocabulary in isiZulu-speaking preschool children

**DOI:** 10.4102/sajcd.v67i1.780

**Published:** 2020-11-19

**Authors:** Xolisile Mazibuko, Moses Chimbari

**Affiliations:** 1 School of Nursing and Public Health, College of Health Sciences, University of KwaZulu-Natal, Durban, South Africa

**Keywords:** schistosomiasis, soil-transmitted helminth, cognitive skills, auditory memory, learning potential, preschool-aged children, isiZulu speakers

## Abstract

**Background:**

This study used local resources- community members, photographer and speech therapists to develop a new test for screening receptive language skills and sought to determine its feasibility for use with a larger population in KwaZulu-Natal province, South Africa.

**Objectives:**

The aim of this study was to develop a one-word receptive vocabulary test appropriate for screening and diagnosis of isiZulu-speaking preschool-aged children. The objectives were (1) to determine sensitivity and specificity of the Ingwavuma Receptive Vocabulary Test (IRVT) and (2) to determine the relationship of IRVT scores with age, gender, time and the confounding variables of stunting and school.

**Method:**

The study was quantitative, cross-sectional and descriptive in nature. The IRVT was piloted before being administered to 51 children (4–6 years old). Statistical analysis of test item prevalence, correlations to confounding variables and validity measurements were conducted using Statistical Package for Social Scientists version 25 (SPSS 25).

**Results:**

The IRVT was able to profile the receptive skills for the preschool children in Ingwavuma. The mean raw score for boys was 35, and 32 for girls. There was a significant Pearson correlation between test scores and age (0.028, *p* < 0.05) with a high effect size (Cohen’s *d* = 0. 949), gender (*r* = –0.032, *p* < 0.05) with a medium effect size (Cohen’s *d* = 0.521) and school (*r* = 0.033, *p* < 0.05) with a small effect size (Cohen’s *d* = 0.353). The sensitivity and specificity values were 66.7% and 33%, respectively. The test reliability (Cronbach’s alpha) was 0.739, with a good test–retest reliability.

**Conclusion:**

The IRVT has potential as a screening test for isiZulu receptive vocabulary skills amongst preschool children. This study contributes to a development of clinical and research resources for assessing language abilities.

## Background

Language is a combination of expressive and receptive communication skills, including the ability to speak, listen, read and write. Language development informs many aspects of child development, including emotional wellness, cognitive abilities and literacy. Information on language development can be sensitive in determining the effects of various medical conditions, such as meningitis, cerebral malaria and human immunodeficiency virus (HIV) (Alcock & Alibhai, 2013). Both expressive and receptive skills are important to understand a child’s overall language development. However, receptive language development precedes language production and ultimately launches expressive abilities, thus building the foundation for the development of literacy (Muller & Brady, 2016).

Receptive language or a child’s ability to understand the language spoken by others and to speak well enough for others to understand them should be achieved by the end of the preschool period (6 years in South Africa), providing the scaffolding for independence and literacy in grade 1 (Committee on the Evaluation of the Supplemental Security Income [SSI] Disability Program for Children with Speech Disorders and Language Disorders, 2016). Language experience in the second year of life can be used to predict language outcomes in late childhood (Gilkerson et al., 2018). Language prediction is important for the development of intervention programmes for supporting parents, preschool teachers and therapists to create optimal learning environments. Strong receptive language skills give preschool children a head start in incremental language processing (Venker, Edwards, & Weismer, 2019). Incremental language processing allows the child, as a listener, to understand pieces of conversation before the utterance is even finished and is a discriminating feature in differential diagnosis of autism spectrum and developmental disorders (Venker et al., 2019). Receptive vocabulary has a significant effect on literacy skills in South African studies (Pretorius & Stoffelsma, 2017; Wilsenach, 2015). Children with severe reading fluency and comprehension problems showed poor receptive language, which lead to problems with expressive language (Frazier, 2011). Hence, when probing into language development and preliteracy skills in preschool children, receptive vocabulary is one of the crucial skills to evaluate.

As much as there is a strong case for obtaining information on children’s receptive language skills and despite available policy frameworks, there is a challenge in gathering such information using formal clinical assessment methods. There are difficulties in developing clinical tests for assessing children using their own home languages taking into account various cultures and multilingualism in South Africa. We acknowledge the known historical challenges in South Africa relating to the paucity of research on language acquisition, limited publications on minority indigenous languages, few therapists who are able to service clients from various indigenous African languages and an overburdened healthcare system (HPCSA, 2019). Thus, not much has been reported on preschool children in relation to language acquisition for clinical purposes to assist with setting realistic therapeutic expectations for isiZulu speakers (Jordaan & Kunene-Nicolas, 2016).

It is therefore imperative that progressive work conducted in isiZulu should be highlighted and lessons learnt from those processes should be used. These include the Zulu Expressive Receptive Language Assessment, which looked into various aspects of morphology, syntax and semantics for preschool children (Bortz, 1995); the problem-solving Test of Ability to Explain for Zulu-speaking children (Solarsh & Alant, 2006); the vocabulary checklist, used mainly in research, for children aged 2–4 years called the Communicative Development Inventory, which has been adapted to various African languages including isiZulu (Alcock et al., 2015); as well as adaptations of language tests into isiZulu, including the Renfrew Action Picture Test (Mdlalo, 2015) and the British Picture Vocabulary Scale (Cockcroft, 2016).

Challenges such as translations influencing sensitivity (Knauer, Karinger, Jakiela, Ozia, & Fernald, 2019), bias and limited comparison to norms (Weber, Fernald, Galasso, & Ratsifandrihamanana, 2015) experienced in test adaptations justify the need to develop local tests especially for isiZulu, the most widely spoken South African language. The aim of our study was to develop a one-word receptive vocabulary test appropriate for screening and diagnosis of isiZulu-speaking preschool children. The objectives were (1) to determine the sensitivity and specificity of the Ingwavuma Receptive Vocabulary Test (IRVT) and (2) to determine the relationship of IRVT scores with age, gender, time and the confounding variables of stunting and school.

**FIGURE 1:**
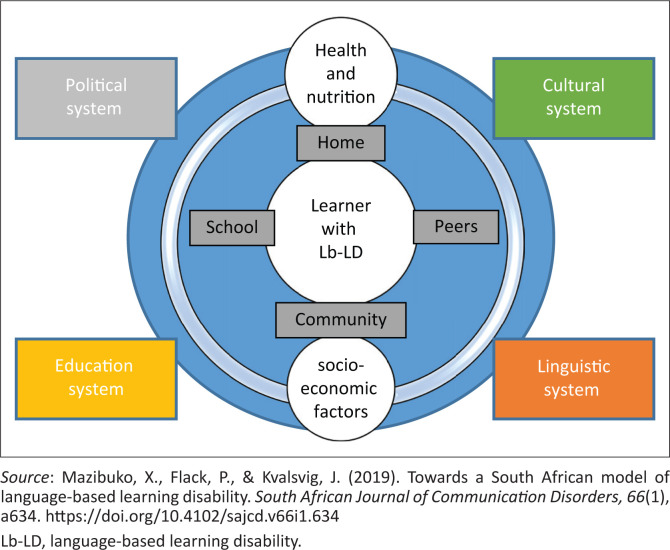
The system of language-based learning disability.

The Bio-ecological Model of Development framed this study considering that a multitude of micro- and macro-systems interact to affect cognitive and language development of any typical child (Bronfenbrenner & Morris, 2007). Since some factors have a distal and some proximal influences on children’s cognitive and language development, our study considered elements from the school-, community- and home-level systems. These include poverty, child-headed homes, poor educational settings and the shortage of water and sanitation (Figure 1). Policy-level issues in the political, educational, health and socio-economic systems affect variability in the linguistic system and have a distal effect on culture and language in the area (Mazibuko, Flack, & Kvalsvig, 2019). Considering that receptive language has an effect on literacy, its impairment may contribute to a language-based learning disability (Lb-LD), a condition highly influenced by genetic or neurological conditions and other systemic imports as indicated in Figure 1.

The interaction of microsystems contributes to not only variations in lateral extensions (variability across all children of a specific age) during development but also in temporal variations (normal variations over time) on language development (McGuinness, 2005).Therefore, socio-economic status (SES) was considered an influencing demographic factor for language development and formed part of risk factors considered in this study as supported by literature on language development in children (Gurgel, Vidor, Joly, & Reppold, 2014). Stunting (low height for age) is an indicator of chronic undernutrition caused by inadequate intake of nutritious foods, linked to households living in poverty and causes irreversible impact on physical and cognitive growth in children (UNICEF, 2020). The effect of stunting on receptive vocabulary was therefore considered in this study because of its direct link with SES and its effect on cognitive development.

We considered that isiZulu-speaking children’s inventory has been linked to linguistic input at home (Kunene-Nicolas & Ahmed, 2016). Therefore, the influence of a child’s background and developmental history on both the quality and quantity of language development are critical to language development (McLaughlin, Sheridan, & Nelson, 2017). Since the risk factors for speech and language impairment include gender, ongoing hearing problems and a reactive temperament whilst protective factors are maternal well-being, biological and psychosocial factors intrinsic to the child, we sought to investigate the effect of gender, age and school on receptive vocabulary (Harrison & McLeod, 2010).

## Study design

**FIGURE 2:**
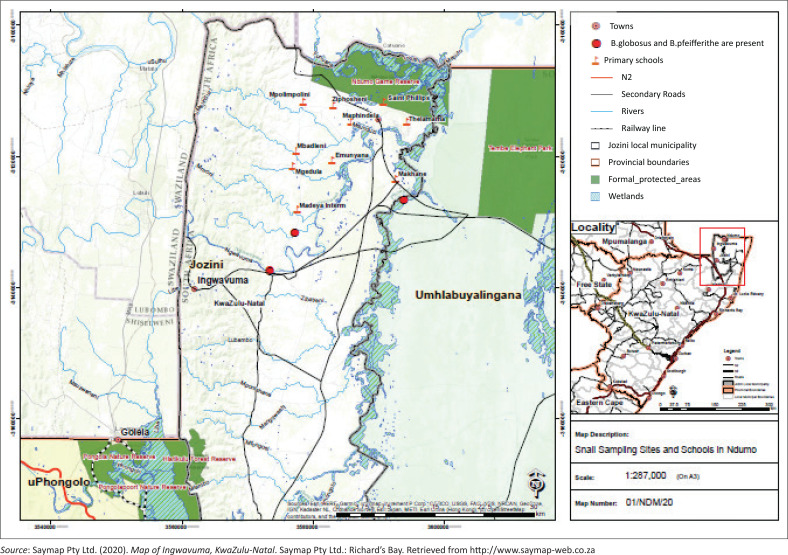
Map showing the location of Ingwavuma in the province of KwaZulu-Natal.

This study was a quantitative, cross-sectional, descriptive study. The study area was Ingwavuma, a small town located in the northern KwaZulu-Natal province (Figure 2). It is close to the borders of eSwatini to the west and Mozambique to the north. It is managed by the Jozini Municipality under the uMkhanyakude district municipality. This municipality is rural, with 89% of the population residing under the jurisdiction of traditional authorities (Statistics South Africa, 2011).

Participants were recruited from 15 preschools and four early childhood development (ECD) centres located in the traditional rural areas of Ndumo, Makwakwa, Manyiseni and Skhemelele within Ingwavuma (red flags in Figure 2). All children above 4 years of age in each preschool were considered for the study, but only those who returned the consent forms and satisfied the inclusion criteria participated. Stratified sampling was used in order to achieve adequate gender and age representation. To be eligible for the study, a child had to be of age between 4.0 and 6.11 years, attend an isiZulu medium preschool or ECD centre in the target area, have no developmental delays, be monolingual isiZulu speaker and pass a hearing screening test.

The government-supported ECD centres in Ingwavuma are located in communities as places of care for children between 3 and 5 years old, operating independently from schools or other non-governmental structures like churches. Based on the teachers’s reports, children between 5 and 6 years, depending on their birthday, are placed in grade R at the nearest preschool attached to a primary school and sent to grade 1 on the year they turn 7 years. The teachers from ECD centres had high school education and were trained locally through workshops organised annually by the Department of Basic Education, whilst teachers placed in preschools attached to a primary school had a basic teaching diploma. The ratio of teacher to students was 1:60 in ECD centres and 1:45 in grade R classes. All preschools that participated had pit toilets and no running tap water. They also had at least one rainwater harvest tank within the facility and provided one meal a day for the children. In our observations on all test dates, the meals did not include green leafy vegetables or meat. Although all schools had government-supplied grade R books, only eight preschools had puzzles or board games and only two schools had a computer for the use of the teacher. None of the preschools had a TV for the children to watch and none had Internet access.

## Procedures

The first step of the assessment process was hearing screening, which entailed otoscopic examination and tympanometry (Harrison & McLeod, 2010). A nutritionist determined the participant’s anthropometric data by calculating body mass index for age (weight in kg) and height for age (height and arm circumference in cm) to determine stunting (World Health Organization, 2011). The children were classified as having: (1) mild, (2) moderate or (3) severe levels of stunting. The prevalence of stunting was 26% with the majority of the participants found to have adequate nutrition. The IRVT was then administered to each child in a separate area or room. Each participant was tested individually by a clinician, in the presence of a community research assistant to make observations of behaviour. The child sat side by side so that both the child and the clinician could see the pictures.

### Data analysis

Data analysis measured Pearson’s chi-square and independent samples T-tests identified relationships and effect size of those correlations between receptive vocabulary scores and dependent variables (Price & Jhangiani, 2013). Multivariate logistic regression model was fitted to identify independent predictors of receptive vocabulary amongst the children. Scale analysis was conducted via Cronbach’s alpha and split half correlations, where a *p*-value less than 0.05 was considered statistically significant. The study was approved by the University of KwaZulu-Natal. Parental consent forms were signed for all participants.

### Developed test description

The IRVT was designed to evaluate receptive vocabulary in isiZulu, including nouns, verbs, adjectives and categories. The blueprint of the test was influenced by typical one-word vocabulary tests (Dunn & Dunn, 2006; Brownwell, 2000). It was essential that the test was developed in conjunction with mother-tongue speakers of the language to increase cultural sensitivity and the development of procedures and items evolved from our pilot study (Holding, Abubakar, & Kitsao-Wekulo, 2010). Appropriate isiZulu words for the age group of 4–6 years were selected by the principal researcher, as a first-language isiZulu speaker and a speech-language therapist with a 20-year practice experience. The word choices were informed by the northern Zululand dialect and isiZulu grammatical structure, which entails a high number of noun classes and a prefixed grammatical morphology, that is, use of vowels as prefixes before nouns and verbs (Magagula, 2009). Code-switching between English and isiZulu is very common in townships and urban areas but not in rural areas or small towns as it is influenced by local culture (Magagula, 2009). However, code-switching was allowed in the IRVT to accommodate learners from different backgrounds and the sound R was included in the word Zebra as this was an acceptable choice of the word in our sample.

High-frequency vocabulary was selected from a South African Curriculum Assessment Policy Statements (CAPS) grade R and grade 1 life skills student book, whilst pictures were collected from public inventory on the Internet (Department of Basic Education, 2019). Words in noun class were the majority in the target list based on the findings that nouns formed the majority of initial words used by toddlers from ethnolinguistically diverse backgrounds in South Africa (Gonasillan, Bornman, & Harty, 2013).

### Pilot study

The first pilot study was conducted in Durban with 10 preschool children from a crèche in Inanda and five PhD students involved in developmental studies. This entailed development of the initial blue print of the test. The second part of the pilot study was conducted on the study site in Ingwavuma in order to finalise the word list adjust for dialectal changes and determine grading of the word list. Observations and experiences of two community research assistants, a second speech therapist and local preschool teachers were used to evaluate the vocabulary choices. The 80% acceptance level was used for inclusion of word choices in the pilot study. The word list was then graded in complexity by the principal researcher, using the grade R and grade 1 CAPS life skills books, with 20 targets at level 1 approximately to 4–5 years of age, 15 at level 2 approximately to ages 5–6 years and 15 at level 3 approximately to 6 years and above. Table 1 displays the target word list, which included 26 Nouns, 8 verbs, 6 adjectives and plan for distractors and illustrations.

**TABLE 1:** Ingwavuma receptive vocabulary test target and illustration plan.

Target	Picture position	English translation	Primary distractor	Secondary distractor	Random picture
1. Isandla	A	Hand	Foot	Hair	Juice
2. Imali	C	Money	Empty wallet	Magazine	Tree
3. Inkomo	B	Cow	Dog	Monkey	Flower
4. Inyoka	D	Snake	Snail	Duck	Grapes
5. Isango/Igate	D	Gate	Door	Car	Shirt
6. Isikebhe	B	Boat	Truck	Water	Apple
7. Uyagibela	C	Climbing (Jungle gym)	Ladder	Stairs	House
8. Isigubhu	A	Drum	Trumpet	Sticks	Cup
9. Iwashi	A	Watch	TV	Sun	Chair
10. Okuphukile	C	Broken (Cup)	Whole eggs	Hammer	Pot
11. Ithoshi	B	Torch	Lamp	Fire	Oil
12. Ufudu	D	Tortoise	Lamb	Rhino	Ball
13. Intamo	D	Neck	Toes	Shoes	Hat
14. Ikhandlela	B	Candle	Torch	Light bulb	Window
15. Inyosi	C	Bee	Bird	Butterfly	Flower
16. Zishayisene	A	Car accident	Cars in traffic	Something falling	Puppy
17. Bayaphana	A	Sharing	2 kids playing	Teacher	Dogs
18. Uyakama	A	Combing	Dressing	Bathing	Walking
19. Inethi	C	Fishing net	Ship	Bicycle	Dress
20. Uyafunda	C	Reading	Baking	Playing	Pencil
21. Isando	B	Hammer	Spanner	Knife	Spoon
22. Ujeke	B	Jug	Teacup	Kettle	Fork
23. Umcibisholo	D	Arrow	Log	Pen	Plate
24. Uyaqoba	D	Chopping	Eating	Working	Jumping
25. Othukile	A	Afraid	Sad	Playing	Walking
26. Uthisha	C	Teacher	Nurse	Grandpa	Children
27. Okubili	B	Two (Balls)	One dot	5 (Digit)	Mountain
28. Izebra/ Idube	D	Zebra	Elephant	Lion	Van
29. Ujabulile	D	Happy	Sad	Chopping	Sleeping
30. Kuyaconsa	B	Leaking	Waterfall	River	Rain
31. Umifino	C	Vegetable	Banana	Meat	Toy
32. Ukudonsisana	A	Pulling	Pushing	Sleeping	TV
33. Izithelo	A	Fruit	Corn	Plant	Ice cream
34. Ingadi	C	Garden	Pot plant	Salad	Airplane
35. Uwuli	B	Wool	Blanket	Bed	Shirt
36. Ophathisiwe	D	Messanger	Envelope	Eating	Sitting
37.Ukubhukuda	D	Swimming	Fishing	Skipping	Sun
38. Iqakala	B	Ankle	Fingers	Tail (Dog)	Train
39. Uboya	C	Fur	Bird	Fish	Toy-(Rattle)
40. Izimpaphe	A	Feathers	Pillow	Grass	Grass
41. Namathisela	A	Sticking	Reading	Writing	Book
42.Idwala	C	Rock	Forest	Bird	Fruit
43. Unesi	C	Nurse	Teacher	Mom	Children
44. Ukhozi	B	Eagle	Duck	Chicken	Motorbike
45. Umpheki	B	Chef	Fireman	Cupcake	Teacher
46.Bayaphikisana	D	Arguing	People smiling	Shaking hands	1 bathing
47. Izimpande	D	Roots	Tree top	Orange	Pear
48. Izinzwane / Intshodo	A	Claw (Chicken)	Hooves	Animal head	Teeth
49. Ihlombe	C	Shoulder	Legs	Hair	Clock
50. Kuqinile	B	Hard	Stone	Bread	River

A 50-page picture book was prepared with four pictures per page, full colour and black-and-white pictures, arranged in graded level of difficulty (Table 1). The illustrations contained one target and three distractors where the primary distractor had to be in the same category; the secondary distractor could be associated with the target and one random picture. The instruction was for the child to point to the correct picture amongst three other distractors. A raw score of one point per target was allocated using a kobo collect app, an open-source platform used for collecting and analysing data (Palla, LeBel, & Chavernac, 2016).

For the purpose of this research, all children were started off in item 1 as a baseline and completed all 50 items bearing no ceiling item. Using floor and ceiling items adjusts a starting level according to age and allows the tester to stop the test when a specific number of errors are made (Weitz, 2008). However, in feasibility studies, using floor and ceiling items is known to reduce the ability to distinguish between children of differing abilities (Prado et al., 2010).

### Test evaluation

The pilot study indicated challenges with picture legibility and vocabulary choices because of the lack of exposure, attention to detail or a combination of distractors. Example of distractor error was the ‘doctor versus nurse’ pictures where the presence of both choices introduced confusion. Similarly, for the target word *ladder*, it included a picture of stairs as a distractor, which led to confusion as the isiZulu word *isi-tebhisi* could apply to both ladder and stairs. The picture of a net resulted in errors because of the lack of familiarity with the western image of a handheld net as opposed to a fishing net. For the target word vegetable or *imifino*, the picture choice was initially a carrot, which turned out to be an unpopular choice as *imifino* usually refers to all green leafy vegetables in isiZulu culture. A picture of spinach was then a solution. Fourteen words matched to easy level with a prevalence between 90% and 100%, whilst moderate had a prevalence between 60% and 80% and 10 difficult items had a prevalence below 50%. These items were not in the expected sequence, implying that some words were more difficult than expected as shown in Appendix 1.

### Ethical consideration

This article followed all ethical standards for a research article.

## Results

### Sample characteristics

**FIGURE 3:**
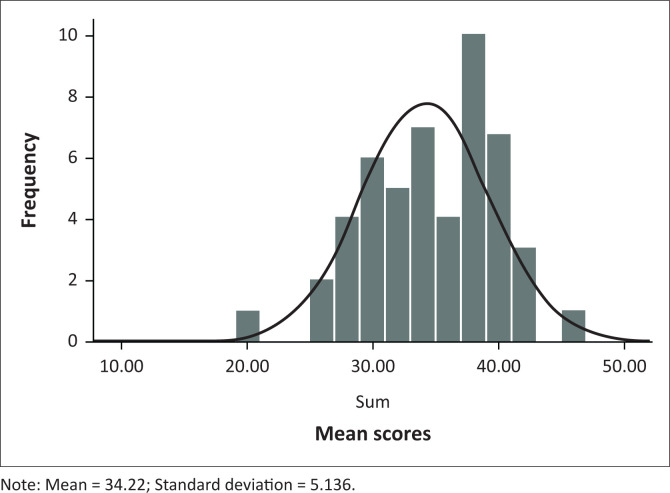
Test performance summary: Ingwavuma receptive vocabulary test, mean scores and distribution (*N* = 50).

The majority of the children fell within the 5-year-old group (mean age = 5.7 years, standard deviation [SD] = 0.852 years). There was less representation of children in the 4-year-old group and above 6.0 years of age. A Shapiro–Wilk’s test (*p* < 0.05), a visual inspection of the histograms, normal Q-Q plots and box plots showed that the test scores were approximately normally distributed for both males and females, with a skewness of -0.401 (standard error [SE] = 0.464) and a kurtosis of −0.288 (*SE* = 0.902) for girls and a skewness of 0.107 (*SE* = 0.464) and a kurtosis of 0.514 (*SE* = 0.902) for boys. The mean for the total raw score was 34 (*SD* = 5.13) and the range was 26. Figure 3 shows the distribution of mean scores, indicating a minimum raw score of 20 and a maximum of 46. The 25th percentile was 30, 50th percentile was 34.5 and 75th percentile was 38.

The results indicate significant differences in performance by gender (*r* = 0.032, 1 tailed *p* < 0.05), which is interesting since gender was equally represented in the sample. The males had a higher mean than females, with a correlation to gender was supported by a moderate Cohen’s *d* (0.521). The results show that children between 4.0 and 5.0 were able to achieve a minimum raw score of 20. Table 2 shows the performance of children in different age bands on the IRVT age bands.

**TABLE 2:** Performance on Ingwavuma receptive vocabulary test by age.

Age band	Mean raw score	*N*	Standard deviation
4.0–4.6	34.5	4	0.0
4.7–5.0	33.1	4	0.0
5.1–5.6	32.3	12	2.1
5.7–6.0	34.9	9	6.7
6.1–6.6	36.6	15	5.2
6.7–7.0	35.0	6	4.2
Total males	35.5	25	4.6
Total females	32.8	25	5.3
Total sample	34.22	50	5.1

The results indicate significant differences in performance by age with Pearson’s correlation of *r* = 0.028 (1 tailed, *p* < 0.05), which was supported by a high effect size as Cohen’s *d* = 0.949. The mean time taken to complete the test (TTT) was 23.14 min, with no correlation of TTT to gender or age.

### Effect of socio-economic factors: Stunting and school

The prevalence of stunting was 26% in our sample and the results showed no significant correlation of test scores to stunting. Bivariate correlation measurement of school attended to test scores was significant as *r* = -0.262 (1 tailed, *p* < 0.05.) and showed a small effect as Cohen’s *d* = 0.353. There was no correlation between school to age and TTT.

### Reliability

Reliability across items was measured through two tests of internal consistency, namely, the Cronbach’s alpha and split half correlation (Price & Jhangiani, 2013). The results indicated adequate Cronbach’s alpha (α = 0.74) and less than ideal Guttman’s s split half coefficient (0.642), whilst the split half range was satisfactory at 0.834 for part 1 and 0.957 for part 2. For analysis of reliability over time, we used test–retest reliability. Eight children (15%) were available for retesting and the results shown in Table 3 indicate a correlation above the ideal of +0.8 and a standard error of measure close to a score of 1.

**TABLE 3:** Test–retest measurements.

Variable	Mean	Standard deviation	Standard error	*N*	*r*	Value	Significance
Trial 1	33.6	3.6	1.28	8	-	-	-
Trial 2	31.75	3.9	1.39	8	-	-	-
Pooled values	-	7.5	2.67	16	-	-	-
Intra-class correlation (Average measure)	-	-	-	-	0.813	6.91	0.010

Note: Standard error measurement = 0.93.

### Validity

A diagnostic test is usually validated against a golden standard that evaluates the same subjects (Wong & Lim, 2011), and in the absence of such an equivalent standard, four measurements of test performance can be used, namely, sensitivity, specificity, positive predictive value (PPV) and negative predictive value (NPV) where the ideal result would be 100% (Wong & Lim, 2011). For our study, the PPV indicated the likelihood that the child who scores well on the IRVT has good receptive vocabulary and the NPV indicated the proportion of the children who have a poor vocabulary amongst those who test negatively on the test. The results showed that five items had both the PPV and NPV less than 50%, thus requiring a review. The IRVT showed an overall sensitivity of 66.7%, reflecting that those who tested positively were true positives, whilst a 33% specificity reflects a correct identification of true negative results or delayed receptive vocabulary.

## Discussion

The IRVT included all possible candidates for assessment whilst aiming for absence of bias in language, equal opportunities, equitable content and use of comprehensive but clear illustrations. Legibility of illustrations (how illustrations and response format have the capability of being deciphered with ease) was ensured though qualitative observations by local people (Johnstone, Altman, Thurlow, & Thompson, 2006). We also monitored item length, avoided words with dual meanings and used high-frequency local vocabulary in the blue print as recommended and these were reflected in the high item prevalence scores (Dempster & Reddy, 2007).

For the analysis of reliability, test items that carried a variance of 1% or 100% score were automatically excluded by the software which negatively influenced the Cronbach’s alpha but the average was still adequate, suggesting that all remaining items measured the same construct. Since there was only one tester, the inter-rater reliability could not be measured; to compensate for this limitation, rigorous analysis of internal consistency and validity were done, which showed satisfactory reliability and sensitivity for language scores.

The TTT mean of 23 min was much higher than the reported mean of 8 min with similar standardised tests (Prado et al., 2018; Weber et al., 2015). The longer TTT can be explained by the lack of basal or floor and ceiling items when the test was administered. By not using floor and ceiling items, the IRVT matched methodological modifications seen in test adaptation studies (Pakendorf & Alant, 1997). Qualitative observation by the tester also indicated extended verbal interaction between the examiner and the children, when establishing rapport, especially the younger participants who contributed to longer TTT. Additionally, in our study, the test was untimed and allowed children enough time to explore the pictures. These are factors that are usually controlled in standardised tests (Educational Psychological Service, 1991). Our findings show, through item prevalence reports, that item grading and test item sequences need adjustments for possible determination of floor and ceiling items with a larger sample.

The prevalence of stunting in our study matched trends in other African countries and similar to other studies conducted in other rural areas of KwaZulu-Natal, where there was a need for additional school resources, poor infrastructure, overcrowded classrooms, a need for inservice training for teachers and a need for more meaningful parental involvement (Hannaway, Govender, Marais, & Meier, 2019). We noted that children from low SES background performed below the normative mean in receptive vocabulary tests, such as the Peabody Picture Vocabulary Test (PPVT) (Allison, Robinson, Hennington, & Bettagere, 2011; Horton-Ikard & Weismer, 2007). Our results showed no correlation between test performance and stunting, probably because of the mild degree of stunting in the sample. We noted a high risk of poverty conditions in the area and that the participant’s home nutrition needs further investigation.

Similarly, the school factor introduced no variation in the performance of children on the IRVT. This similarity does not reduce the significance of considering the children’s context and bio-psycho-social factors when assessing language. Factors such as teacher experience, level of skills, play behaviour and learning resources have been proven to directly impact learning outcomes, whilst factors like water source and sanitation contribute to cultural variations within societies (Feza, 2018; Spencer et al., 2017). The performance of the children on this test is comparable to other studies in terms of the age effect on the vocabulary (Kunene-Nicolas & Ahmed, 2016). The results are unique in that boys performed better than girls, whilst being male has been associated with risk factors of speech and language impairment (Harrison & McLeod, 2010).

## Limitations of the study

The IRVT needs to be evaluated with a larger sample size for evaluation of the adjusted word list, illustrations and for generalisability of the results. Although the IRVT achieved moderate sensitivity, there is an indication from the PPV and NPV that adjustments to the vocabulary choice, grading and presentation order of items should provide better results. Furthermore, the test item selection was based on the opinions of experienced adult language users and CAPS books. We acknowledge the subjectivity associated with the process and the technical exclusion of the 4-year-old group that was not the target for the books. Another limitation of the study was a small sample size and a lack of an in-depth individualised socio-economic questionnaire which could have contextually ascertained the level of risk for speech and language delays (Hoff, 2013). The strength of the IRVT is that it has a single purpose and does not require in-depth training.

## Conclusion

Receptive language lays the foundation for expressive language development and allows for scaffolding of literacy skills, such as reading and writing. A test of receptive vocabulary in isiZulu is a valuable tool in baseline school readiness assessment of isiZulu-speaking preschool children to achieve early intervention. The children in this study took an average of 23 min to complete the one-word receptive vocabulary test in isiZulu and obtained an average score of 35, with boys performing statistically better than girls, whilst a significant variation in scores depended on age and school attended. The IRVT is a promising screening tool appropriate for preschool children from low SES communities. However, there is a need to adjust the test structure, evaluate it using a larger sample size, develop a complimentary intervention programme and align it with educational goals.

## Appendix 1

### Additional validity data

**TABLE 1-A1:** Ingwavuma receptive vocabulary scale form.

Target	Level grading	Initial grade	Prevalence (%)
1. Isandla	1	Easy	100.0
2. Imali	1	Easy	100.0
3. Inkomo	1	Easy	96.0
4. Inyoka	1	Easy	100.0
5. Isango/ igate	1	Medium	86.3
6. Isikebhe	1	Medium	100.0
7. Uyagibela	1	Medium	62.0
8. Isigubhu	1	Medium	98.0
9. Iwashi	1	Medium	98.0
10. Okuphukile	1	Medium	86.3
11. Ithoshi	1	Medium	96.0
12. Ufudu	1	Difficult	100.0
13. Intamo	1	Difficult	86.3
14. Ikhandlela	1	Difficult	100.0
15. Inyosi	1	Difficult	94.0
16. Ingozi	1	Difficult	82.4
17. Bayaphana	1	Difficult	78.4
18. Uyakama	1	Difficult	98.0
19. Inethi	1	Difficult	56.9
20. Uyafunda	1	Difficult	98.0
21. Isipanela	2	Easy	88.2
22. Ujeke	2	Easy	76.5
23. Umcibisholo	2	Easy	90.2
24. Uyakabha	2	Easy	78.4
25. Othukile	2	Easy	82.4
26. Uthisha	2	Medium	70.6
27. Okubili	2	Medium	52.9
28. Izebra/ Idube	2	Difficult	27.5
29. Ujabulile	2	Difficult	37.3
30. Kuyaconsa	2	Difficult	37.7
31. Umifino	2	Difficult	54.9
32. Bayadonsisana	2	Difficult	70.6
33. Izithelo	3	Easy	35.3
34. Ihlathi	3	Easy	96.0
35. Uwuli	3	Easy	33.3
36. Ophathisiwe	3	Medium	52.9
37. Ukutshuza	3	Medium	60.8
38. Iqakala	3	Medium	25.5
39. Uboya	3	Medium	66.7
40. Uphaphe	3	Medium	23.5
41. Uyanamathisela	3	Medium	43.1
42. iIwala	3	Medium	47.1
43. Udokotela	3	Medium	60.8
44. Ukhozi	3	Difficult	41.2
45. Umpheki	3	Difficult	70.6
46. Bayaphikisana	3	Difficult	62.7
47. Izimpande	3	Difficult	19.2
48. Izinzwane/Intshodo	3	Difficult	82.4
49. Isihlakala	3	Difficult	7.8
50. Uketshezi	3	Difficult	17.6

**TABLE 2-A1:** Test item-specific validity measurements.

Test item	Sensitivity (%)	Specificity (%)	PPV (%)	NPV (%)
1.	66.7	33.3	100.0	100.0
2.	66.7	33.3	100.0	100.0
3.	66.7	33.3	100.0	100.0
4.	68.0	100.0	100.0	5.9
5.	68.0	100.0	100.0	5.9
6.	63.6	14.3	82.4	5.9
7.	66.7	33.3	100.0	100.0
8.	66.7	33.3	100.0	100.0
9.	66.0	0.0	97.1	0.0
10.	71.9	42.1	67.6	47.1
11.	85.7	40.5	35.3	88.2
12.	67.3	50.0	97.1	5.9
13.	65.9	28.6	85.3	11.8
14.	66.7	33.3	100.0	100.0
15.	64.6	0.0	91.2	0.0
16.	61.9	11.1	76.5	5.9
17.	58.3	30.8	20.6	70.6
18.	68.0	100	100.0	5.9
19.	62.1	27.3	52.9	35.3
20.	64.6	0.0	93.9	0.0
21.	62.2	0.0	82.4	0.0
22.	70.5	57.1	91.2	23.5
23.	67.4	40.0	91.2	11.8
24.	67.6	35.7	73.5	29.4
25.	67.5	36.4	79.4	23.5
26.	83.9	60.0	76.5	70.6
27.	61.0	11.1	76.5	5.9
28.	64.3	22.2	79.4	11.8
29.	67.6	35.3	67.6	35.3
30.	61.1	20.0	64.7	17.6
31.	44.8	28.6	11.8	70.6
32.	67.3	50.0	97.1	5.9
33.	47.6	20.0	29.4	35.3
34.	59.1	27.6	38.2	47.1
35.	76.5	38.2	38.2	76.5
36.	67.9	34.8	55.9	47.1
37.	58.1	20.0	52.9	23.5
38.	69.2	34.2	26.5	76.5
39.	74.4	58.3	85.3	41.2
40.	47.4	21.9	26.5	41.2
41.	72.2	46.7	76.5	41.2
42.	66.7	33.3	47.1	52.9
43.	69.4	40.0	73.5	35.3
44.	77.8	39.4	41.2	76.5
45.	68.4	34.4	38.2	64.7
46.	65.6	31.6	61.8	35.3
47.	80.0	36.6	23.5	88.2
48.	59.3	25.0	47.1	35.3
49.	75.0	34.0	8.8	94.1
50.	63.0	29.2	50.0	41.0
Total	66.7	33.3	-	-

PPV, positive predictive value; NPV, negative predictive value.
